# The inflammation-fibrosis combined index as a predictor of prognosis in stroke patients undergoing mechanical thrombectomy

**DOI:** 10.3389/fnagi.2026.1795011

**Published:** 2026-04-29

**Authors:** Xiaoping Xu, Mengting Chen, Yaojia Xu, Xia Chang, Yongxin Li, Yunnan Lu, Yumin Liu

**Affiliations:** 1 Department of Neurology, Xishan People's Hospital of Wuxi City, Wuxi, Jiangsu, China; 2 Department of Neurology, The Affiliated Zhangjiagang Hospital of Soochow University, Suzhou, Jiangsu, China; 3 Department of Neurology, Second People's Hospital of Wuxi City, Wuxi, Jiangsu, China

**Keywords:** endovascular treatment, fibrosis-4, inflammation-fibrosis combined index, ischemic stroke, systemic immune-inflammation index

## Abstract

**Background and purpose:**

This study evaluates the novel Inflammation-Fibrosis Combined Index (IFCI) for predicting 90-day functional outcomes post-MT.

**Methods:**

We conducted a retrospective analysis of 548 consecutive acute ischemic stroke patients treated with MT. The IFCI was derived from the combination of the Fibrosis-4 (FIB-4) index and the systemic immune-inflammation index (SII). The primary outcome was an unfavorable functional outcome (modified Rankin Scale score 3–6) at 90 days. The association between IFCI and the outcome was assessed using multivariable logistic regression and restricted cubic splines.

**Results:**

Among the 548 patients (mean age 70.6 years, 56.2% male), 275 (50.2%) experienced an unfavorable outcome. In multivariable analysis, each 1-point increase in IFCI was independently associated with a 2.51-fold increased odds ratio of poor outcome (95% confidence interval [CI], 1.69–3.73; *P* = 0.001). Patients in the highest IFCI quartile had an 8.79-fold increased odds ratio compared to those in the lowest quartile (95% CI, 3.574–21.661; *P* = 0.001). The IFCI demonstrated superior discriminative ability (area under the curve [AUC] = 0.725) compared to FIB-4 (AUC = 0.683, *P* = 0.012) and SII (AUC = 0.604, *P* = 0.001). Adding IFCI to a model containing conventional risk factors significantly improved risk reclassification (continuous net reclassification improvement = 0.533, integrated discrimination improvement = 0.065; both *P* = 0.001).

**Conclusions:**

By integrating the marker of inflammation and fibrosis pathways, the IFCI is an independent predictor of 90-day outcome after MT. It provides prognostic value beyond conventional variables and its individual components, and may serve as a useful tool for early risk assessment and patient management.

## 1 Introduction

Stroke represents a major global health challenge, being one of the leading causes of death global mortality and long-term disability (Saini et al., 2021; Ji et al., 2025; Ma et al., 2021). The advent of mechanical thrombectomy (MT) has revolutionized treatment for large vessel occlusion, significantly improving recanalization rates and functional outcomes (Goyal et al., 2016; Makkawi et al., 2024). Despite these advances, a considerable proportion of patients still experience unfavorable functional recovery even after successful reperfusion, a phenomenon often termed “futile recanalization” (Wang and Xiong, 2023; Nie et al., 2023; Dhillon et al., 2023). This underscores the urgent need for reliable early prognostic tools to identify high-risk individuals, optimize post-procedural care, and guide personalized rehabilitation strategies.

Emerging evidence underscores the pivotal role of systemic inflammation and its interplay with fibrotic pathways in shaping ischemic brain injury and repair (Wang et al., 2022; Garcia-Bonilla et al., 2025; Huang et al., 2024). Post-stroke inflammation can exacerbate secondary neuronal damage and disrupt the neurovascular unit (Iordache et al., 2025; Wang et al., 2025). Furthermore, activation of fibrotic mechanisms, often reflected in hepatic fibrosis indices, may indicate a maladaptive systemic response and compromised organ reserve, potentially hindering neurological recovery (Zhao et al., 2022). The Fibrosis-4 (FIB-4) index, a marker of hepatic fibrosis derived from routine liver enzymes and platelet count, and the systemic immune-inflammation index (SII), integrating neutrophil, lymphocyte, and platelet counts, have independently been associated with adverse outcomes in cerebrovascular diseases (Xu et al., 2024; Shi et al., 2025; Adiguzel et al., 2022). We hypothesize that a single metric combining inflammation and fibrosis may offer superior prognostic insight. The Inflammation-Fibrosis Combined Index (IFCI), integrating the FIB-4 and SII, has recently demonstrated strong predictive value for left ventricular reverse remodeling and prognosis in heart failure with reduced ejection fraction (Shi et al., 2024), suggesting its utility in conditions marked by inflammatory and fibrotic remodeling. However, its application in the context of ischemic stroke, particularly following MT, remains entirely unexplored.

Therefore, this study aimed to investigate the relationship between the IFCI and 90-day functional outcomes in acute ischemic stroke patients treated with MT, and to evaluate the predictive superiority of the IFCI compared to the SII and FIB-4 indices for poor prognosis.

## 2 Materials and methods

### 2.1 Study sample

This retrospective study analyzed ischemic stroke patients with anterior circulation large vessel occlusion who underwent MT at July 2021 to June 2025 at the stroke centers (Center 1: Xishan People's Hospital of Wuxi City; Center 2: The Affiliated Zhangjiagang Hospital of Soochow University; Center 3: Second People's Hospital of Wuxi City. The inclusion criteria were: (1) adults aged ≥18 years; (2) occlusion of the internal carotid artery or middle cerebral artery. The exclusion criteria were: (1) lack of laboratory data required for IFCI calculation; (2) a pre-stroke modified Rankin Scale (mRS) score > 2; (3) concurrent diagnosis of inflammatory diseases, cancer, immune disorders, or pre-existing chronic liver or kidney disease. The study was performed in accordance with the principles of the Declaration of Helsinki and received approval from the Institutional Review Board at each participating center. Given its retrospective design, the requirement for informed consent was waived by the Institutional Review Board.

### 2.2 Baseline data collection

Demographic information, vascular risk factors, clinical presentations, and laboratory parameters were collected for all patients. Trained neurologists assessed baseline stroke severity using the NIHSS (Brott et al., 1989). We measured pre-treatment infarct volume with the Alberta Stroke Program Early Computed Tomography (ASPECT; Nagel et al., 2018), and determined stroke etiology via the Trial of Org 10,172 in Acute Stroke Treatment (TOAST) classification (Adams et al., 1993). Collateral status was graded per the ASITN/SIR system based on angiographic findings, a grade of 0–1 reflected poor collaterals, whereas 2–4 indicated moderate-to-excellent status (Zaidat et al., 2013). In addition, successful recanalization was defined as achieving a modified Thrombolysis in Cerebral Infarction score of 2b or 3 (Zhang et al., 2020, 2021).

### 2.3 Measurement of IFCI

Blood samples were obtained after EVT, usually within 24 h of stroke onset. Laboratory tests, including complete blood count and liver function tests. SII = neutrophil count (10^3^/μL) × platelet count (10^3^/μL)/lymphocyte count. FIB-4 index = (age × AST) / (platelet count × ALT^∧^0.5). The IFCI was calculated using the formula based on the multivariate logistic regression model: IFCI = β0 + β1 × FIB-4 + β2 × SII. In this study, the derived formula for IFCI was: IFCI = −2.200 + (0.639 × FIB-4) + (0.001 × SII).

### 2.4 Assessment of outcome

All participants were evaluated with the mRS score by independent neurologists blinded to the clinical data. At 90 days after MT, clinicians evaluated patients via telephone or outpatient follow-up to inquire about their self-care abilities. In this study, unfavorable outcome was defined as an mRS score of 3–6.

### 2.5 Statistical analysis

Continuous variables were presented as mean±standard deviation (SD) if normally distributed or as median (interquartile range, IQR) if non-normally distributed, and were compared using the Student's *t*-test or Mann–Whitney *U* test as appropriate. Categorical variables were expressed as frequencies and compared using the chi-square test or Fisher's exact test. To assess the independent association between IFCI and the clinical outcome, binary logistic regression models were constructed: Model 1 was adjusted for age and sex, while Model 2 included demographic characteristics, diabetes, ischemic heart disease, baseline NIHSS score, baseline ASPECT score, stroke centers, stroke etiology, occlusion site, poor collateral status, successful reperfusion, and levels of Hs-CRP and baseline glucose. The IFCI was analyzed both as a continuous variable (per 1-score increment) and as categorical variable (quartiles). The results were reported as odds ratio (OR) with 95% confidence intervals (CI). We also used the restricted cubic spline regression with 3 knots (located at the 5th, 50th, and 95th percentiles) was applied to characterize the association between IFCI and the log odds of poor outcome (Durrleman and Simon, 1989). Furthermore, to quantify the improvement in risk prediction after adding IFCI to conventional risk factors, continuous net reclassification improvement (NRI) and integrated discrimination improvement (IDI) were calculated along with their 95% CI.

Furthermore, the discriminative performance of IFCI, FIB-4, and SII for predicting the 90-day unfavorable outcome was evaluated using receiver operating characteristic (ROC) curves, and the areas under the curves (AUC) were compared by DeLong's test. All statistical analyses were performed using SPSS version 25.0 (IBM Corp., Armonk, NY, USA) and R software (version 4.3.1; R Foundation for Statistical Computing, Vienna, Austria). A two-tailed *P* value < 0.05 was considered statistically significant.

## 3 Results

Finally, a total of 548 stroke patients (56.2% male) who underwent MT were included in the study. The mean age was 70.6 years. Among these patients, 76.8% had hypertension, 42.0% had diabetes mellitus, 10.8% had hyperlipidemia. The median NIHSS score was 14 (IQR 10–18) at hospital admission. The median baseline ASPECT score was 9 (IQR 8–9). Successful reperfusion represented 91.6% of all cases, and 36.1% of patients received intravenous thrombolysis before MT. Baseline characteristics of the patients stratified by quartiles of the IFCI were presented in Table 1. Age (*P* = 0.001), hypertension (*P* = 0.006), diabetes mellitus (*P* = 0.003), baseline NIHSS score (*P* = 0.001), baseline ASPECT score (*P* = 0.033) and baseline glucose levels (*P* = 0.016) differed significantly with increasing quartiles of IFCI.

**Table 1:** Baseline characteristics of the study sample stratified by the quartile of IFCI.

Variables	1st quartile (*n* = 138)	2nd quartile (*n* = 139)	3rd quartile (*n* = 134)	4th quartile (*n* = 137)	*P* value
Demographic characteristics					
Age, year	62.1 ± 10.5	70.3 ± 9.2	74.3 ± 8.3	75.8 ± 9.9	0.001
Male, *n* (%)	74 (53.6)	79 (56.8)	73 (54.5)	82 (59.9)	0.729
Medical history, *n* (%)					
Hypertension	91 (65.9)	112 (80.6)	109 (81.3)	109 (79.6)	0.006
Diabetes mellitus	46 (33.3)	60 (43.2)	50 (37.3)	74 (54.0)	0.003
Hyperlipidemia	17 (12.3)	12 (8.6)	12 (9.0)	18 (13.1)	0.519
Ischemic heart disease	15 (10.9)	23 (16.5)	26 (19.4)	28 (20.4)	0.143
Smoking	46 (33.3)	46 (33.1)	53 (39.6)	46 (33.6)	0.630
Clinical data					
Systolic blood pressure, mmHg	136.9 ± 20.7	137.8 ± 24.0	139.6 ± 23.0	139.0 ± 22.8	0.762
Diastolic blood pressure, mmHg	85.0 ± 12.4	85.6 ± 14.2	82.6 ± 13.4	83.4 ± 15.5	0.303
Baseline NIHSS score	12.0 (8.0, 15.0)	14.0 (10.0, 18.0)	14.0 (12.0, 18.0)	16.0 (12.0, 21.0)	0.001
Baseline ASPECT score	9.0 (8.0, 9.0)	9.0 (8.0, 10.0)	9.0 (8.0, 9.0)	8.0 (8.0, 9.0)	0.033
Time from onset to blood draw, h	18.5 (12.0, 24.0)	19.0 (12.5, 23.0)	19.5 (14.0, 23.0)	18.5 (13.0, 24.0)	0.956
Time from onset to reperfusion, min	403.0 (226.0, 694.0)	345.0 (239.0, 515.5)	340.0 (247.0, 511.0)	320.0 (233.0, 491.0)	0.286
Stroke causes, *n* (%)					0.205
LAA	68 (49.3)	68 (48.9)	62 (46.3)	70 (51.1)	
Cardioembolic	52 (37.7)	59 (42.4)	66 (49.3)	54 (39.4)	
Undetermined or others	18 (13.0)	12 (8.6)	6 (4.5)	13 (9.5)	
Occlusion site, *n* (%)					0.406
Internal carotid artery	46 (33.3)	55 (39.6)	50 (37.3)	59 (43.1)	
Middle cerebral artery	92 (66.7)	84 (60.4)	84 (62.7)	78 (56.9)	
Prior intravenous thrombolysis, *n* (%)	40 (29.0)	53 (38.1)	55 (41.0)	50 (36.5)	0.195
Poor collateral vessels, *n* (%)	67 (48.6)	70 (50.4)	77 (57.5)	67 (48.9)	0.421
Successful reperfusion, *n* (%)	128 (92.8)	127 (91.4)	121 (90.3)	126 (92.0)	0.904
Laboratory data					
Baseline glucose levels, mmol/L	6.7 ± 2.1	7.6 ± 2.7	7.2 ± 2.5	7.4 ± 2.4	0.016
Hs-CRP, mg/L	8.8 (3.7, 19.3)	12.3 (4.1, 30.6)	12.3 (5.0, 35.6)	14.9 (5.1, 30.5)	0.166
FIB-4	1.16 (0.89, 1.51)	1.82 (1.50, 2.25)	2.49 (1.93, 3.02)	3.60 (2.65, 4.89)	0.001
SII	917.7 (619.5, 1,279.8)	1,381.1 (853.8, 1,936.3)	1,661.6 (1,048.3, 2,515.9)	2,213.8 (1,187.9, 3,557.5)	0.001

ASPECT, Alberta stroke program early CT; FIB-4, fibrosis-4 index; Hs-CRP, hyper-sensitive C-reactive protein; IFCI, inflammation-fibrosis combined index; LAA, large arterial atherosclerosis; NIHSS, national institutes of health stroke scale; SII, systemic immune inflammation index.

During the 90 days follow-up, 275 patients were observed with unfavorable outcome. The comparison of baseline data according to patients with and without unfavorable outcome at 90 days were demonstrated in Table 2. Patients with poor outcome were older (mean 73.9 vs. 67.2 years, *P* = 0.001) and had a higher prevalence of diabetes mellitus (49.1% vs. 34.8%, *P* = 0.001) and ischemic heart disease (20.0% vs. 13.6%, *P* = 0.043). They also presented with higher baseline NIHSS scores (median 16.0 vs. 12.0, *P* = 0.001), lower ASPECT score (median 8.0 vs. 9.0, *P* = 0.001), higher systolic blood pressure (mean 140.4 vs. 136.2 mmHg, *P* = 0.028), and were more likely to have an internal carotid artery occlusion (45.1% vs. 31.5%, *P* = 0.001) and poor collateral vessels (55.6% vs. 46.9%, *P* = 0.040). Successful reperfusion was less frequent in the unfavorable outcome group (85.6% vs. 97.4%, *P* = 0.001). Laboratory findings also differed significantly: patients with unfavorable outcomes had higher levels of baseline glucose (mean, 7.8 vs. 6.7 mmol/L, *P* = 0.001), Hs-CRP (median, 14.9 vs. 8.6 mg/L, *P* = 0.001), FIB-4 (median 2.36 vs. 1.67, *P* = 0.001), SII (median, 1,582.6 vs. 1,184.2, *P* = 0.001), and IFCI values (median, 0.15 vs. −0.39, *P* = 0.001).

**Table 2:** Comparison of baseline data according to patients with and without unfavorable outcome at 90 days.

Variables	Unfavorable outcome at 90 days		*P* value
	Yes (*n* = 275)	No (*n* = 273)	
Demographic characteristics			
Age, year	73.9 ± 9.7	67.2 ± 10.9	0.001
Male, *n* (%)	147 (53.5)	161 (59.0)	0.193
Medical history, *n* (%)			
Hypertension	217 (78.9)	204 (74.7)	0.246
Diabetes mellitus	135 (49.1)	95 (34.8)	0.001
Hyperlipidemia	32 (11.6)	27 (9.9)	0.510
Ischemic heart disease	55 (20.0)	37 (13.6)	0.043
Smoking	93 (33.8)	98 (35.9)	0.610
Clinical data			
Systolic blood pressure, mmHg	140.4 ± 22.9	136.2 ± 22.1	0.028
Diastolic blood pressure, mmHg	84.7 ± 14.1	83.4 ± 13.8	0.263
Baseline NIHSS score	16.0 (13.0, 22.0)	12.0 (8.0, 15.0)	0.001
Baseline ASPECT score	8.0 (8.0, 9.0)	9.0 (8.0, 10.0)	0.001
Time from onset to blood draw, h	19.0 (13.0, 24.0)	18.5 (13.0, 23.0)	0.768
Time from onset to reperfusion, min	345.5 (241.5, 558.5)	340.5 (230.0, 548.0)	0.825
**Stroke causes**, ***n*** **(%)**			0.141
LAA	139 (50.5)	129 (47.3)	
Cardioembolic	118 (42.9)	113 (41.4)	
Undetermined or others	18 (6.5)	31 (11.4)	
**Occlusion site**, ***n*** **(%)**			0.001
Internal carotid artery	124 (45.1)	86 (31.5)	
Middle cerebral artery	151 (54.9)	187 (68.5)	
Prior intravenous thrombolysis, *n* (%)	101 (36.7)	97 (35.5)	0.771
Poor collateral vessels, *n* (%)	153 (55.6)	128 (46.9)	0.040
Successful reperfusion, *n* (%)	236 (85.6)	266 (97.4)	0.001
Laboratory data			
Baseline glucose levels, mmol/L	7.8 ± 2.5	6.7 ± 2.3	0.001
Hs-CRP, mg/L	14.9 (5.5, 37.0)	8.6 (3.1, 20.0)	0.001
FIB-4	2.36 (1.67, 3.37)	1.67 (1.15, 2.36)	0.001
SII	1,582.6 (916.7, 2,587.0)	1,184.2 (730.0, 1,852.6)	0.001
IFCI	0.15 (-0.36, 0.92)	−0.39 (-0.83, 0.03)	0.001

ASPECT, Alberta stroke program early CT; FIB-4, fibrosis-4 index; Hs-CRP, hyper-sensitive C-reactive protein; IFCI, inflammation-fibrosis combined index; LAA, large arterial atherosclerosis; MCA, middle cerebral artery; NIHSS, national institutes of health stroke scale; SII, systemic immune inflammation index.

**Figure 1:**
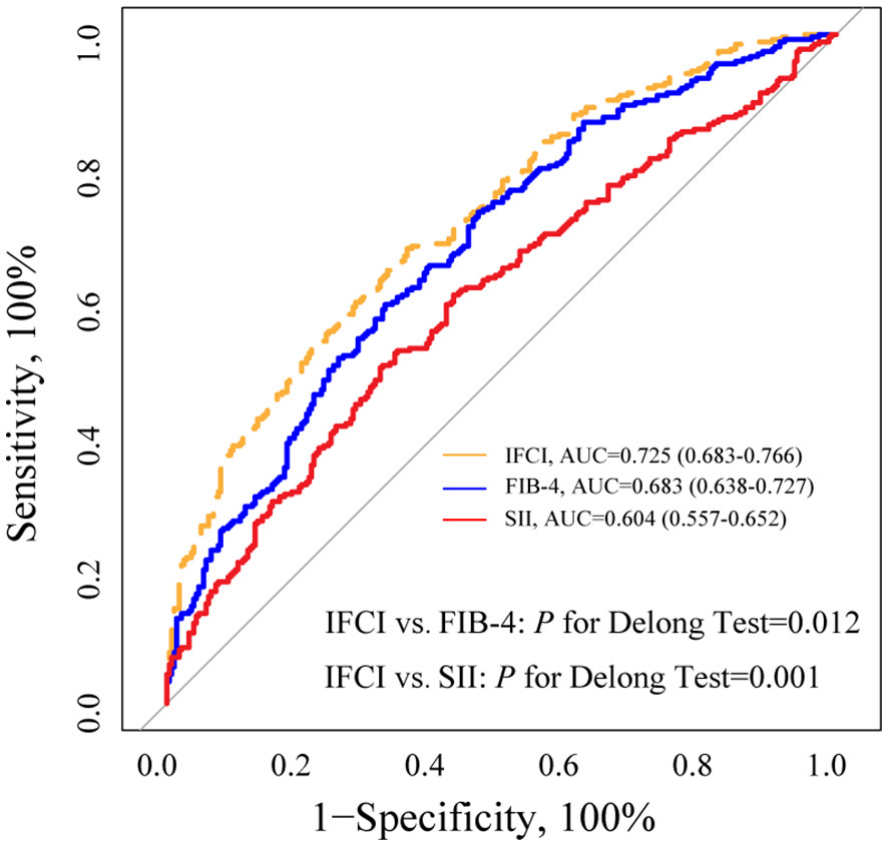
Spline curve plotting baseline IFCI against the ratio odds ratio of poor prognosis. Association was fitted with spline curve with 3 knots (at fifth, 50th, and 95th percentiles) adjusted for covariates included in model 2 in Table 3. Abbreviations: The solid line represented the odds ratio and the dashed lines represented the 95% confidence interval. Abbreviations: CI, Confidence interval; OR, Odds ratio.

Table 3 summarized the results of the binary logistic regression for the association between IFCI levels and risk of poor outcome at 90 days. In the unadjusted model, each 1-score increase in IFCI was associated with an OR of 2.959 (95% CI, 2.285–3.832; *P* = 0.001). After adjusting for demographic and clinical confounders in Model 2, the association remained significant (OR, 2.510; 95% CI, 1.690–3.728; *P* = 0.001). When analyzed by quartiles, the highest IFCI quartile was independently associated with an 8.79-fold increased risk of unfavorable outcome (95% CI, 3.574–21.661; *P* = 0.001) compared to the lowest quartile. Restricted cubic splines further illustrated the dose–response relationship between baseline IFCI and the log odds of poor prognosis (*P* for linearity = 0.001, Figure 1), after adjustment for covariates. The addition of IFCI to a model containing conventional risk factors significantly improved risk reclassification poor outcome (category- continuous NRI, 0.533; 95% CI, 0.375–0.694, *P* = 0.001; IDI, 0.065, 95% CI, 0.045–0.086, *P* = 0.001; Table 4).

**Table 3:** Logistic regression analysis for the association between IFCI levels and risk of poor outcome at 90 days.

Variables	Unadjusted model		Model 1		Model 2	
	OR (95% CI)	*P* value	OR (95% CI)	*P* value	OR (95% CI)	*P* value
IFCI, Per 1-score increase	2.959 (2.285–3.832)	0.001	2.442 (1.866–3.196)	0.001	2.510 (1.690–3.728)	0.001
IFCI, quartile						
1st	Reference		Reference		Reference	
2nd	2.638 (1.577–4.411)	0.001	2.032 (1.183–4.489)	0.001	1.759 (0.795–3.893)	0.163
3rd	3.659 (2.201–6.203)	0.001	2.470 (1.404–4.364)	0.002	3.341 (1.430–7.806)	0.005
4th	11.348 (6.463–19.972)	0.001	7.502 (4.076–13.809)	0.001	8.789 (3.574–21.661)	0.001

CI, confidence interval; IFCI, inflammation-fibrosis combined index; OR, odds ratio.

Model 1: adjusted for demographic characteristics; Model 2: adjusted for demographic characteristics, diabetes, ischemic heart disease, baseline NIHSS score, baseline ASPECT score, stroke centers, stroke etiology, occlusion site, poor collateral status, successful reperfusion, and levels of Hs-CRP and baseline glucose.

**Table 4:** Reclassification statistics (95% CI) for poor outcome after adding IFCI levels.

Models	NRI (Continuous)		IDI	
	Estimate (95% CI)	*P* value	Estimate (95% CI)	*P* value
Model 1				
+ IFCI levels (per 1-score increase)	0.520 (0.362–0.678)	0.001	0.090 (0.066–0.114)	0.001
+ IFCI levels (quartile)	0.548 (0.389–0.709)	0.001	0.074 (0.052–0.096)	0.001
Model 2				
+ IFCI levels (per 1-score increase)	0.533 (0.375–0.694)	0.001	0.065 (0.045–0.086)	0.001
+ IFCI levels (quartile)	0.490 (0.328–0.654)	0.001	0.046 (0.029–0.063)	0.001

CI, confidence interval; IDI, integrated discrimination improvement; IFCI, Inflammation-fibrosis combined index; NRI, net reclassification improvement.

Model 1: adjusted for demographic characteristics; Model 2: adjusted for demographic characteristics, diabetes, ischemic heart disease, baseline NIHSS score, baseline ASPECT score, stroke centers, stroke etiology, occlusion site, poor collateral status, successful reperfusion, and levels of Hs-CRP and baseline glucose.

**Figure 2:**
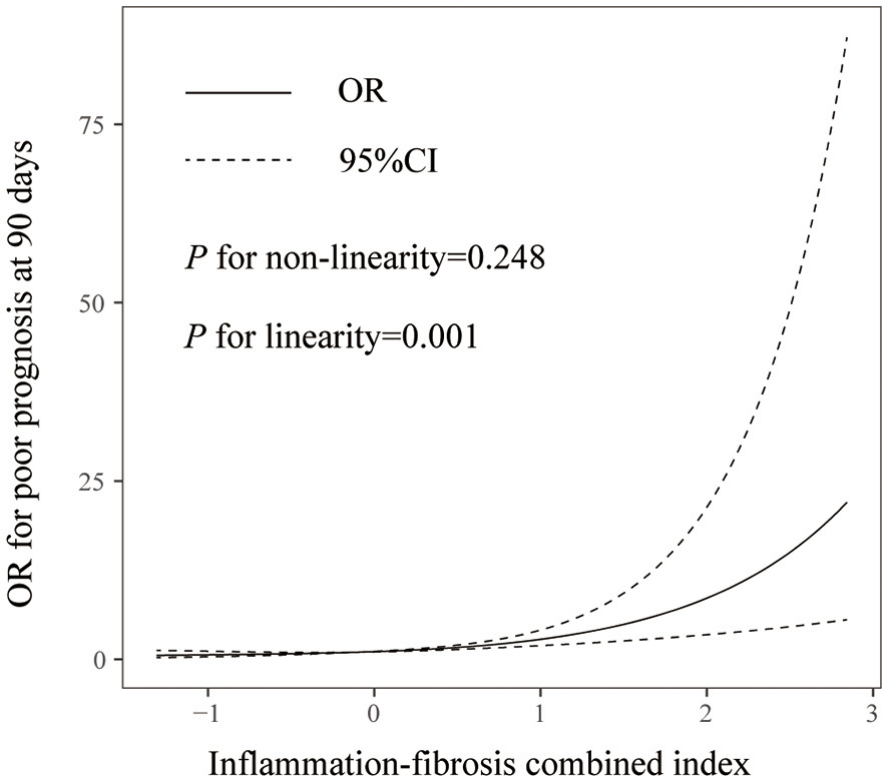
Receiver operating characteristic curves for IFCI, FIB-4, and SII in predicting the incidence of poor outcome at 90 days after mechanical thrombectomy. Abbreviations: AUC, area under the curve; FIB-4, fibrosis-4 index; IFCI, Inflammation-fibrosis combined index; SII, Systemic immune inflammation index.

In addition, as shown in Figure 2, the IFCI score (AUC = 0.725) demonstrated significantly superior discriminative performance for predicting the 90-day poor outcome compared to both the FIB-4 index (AUC = 0.683; *P* = 0.012, DeLong's test) and the SII (AUC = 0.604; *P* = 0.001, DeLong's test).

## 4 Discussion

This study assessed the relationship between IFCI at admission and clinical outcomes in 548 patients receiving endovascular treatment for acute ischemic stroke. To maintain cohort homogeneity and strengthen the internal validity of the results, the analysis was restricted to anterior circulation strokes, as the evidence supporting MT efficacy is strongest for this subgroup (Goyal et al., 2016). We demonstrated that higher IFCI values were significantly associated with an increased risk of unfavorable outcome, even after comprehensive adjustment for established clinical, imaging, and laboratory confounders. Notably, the IFCI exhibited superior discriminative performance compared to its individual components, and significantly improved risk reclassification when added to conventional prognostic models.

Reperfusion occurs following the recanalization of an occluded vessel and the restoration of antegrade blood flow. Our data demonstrated that successful recanalization correlated with decreased rates of unfavorable outcome, confirming and extending prior evidence of its positive outcome impact despite reperfusion injury risks (Winkelmeier et al., 2023). Established risk factors such as advanced age and a lower baseline ASPECT score were also reaffirmed in our data as predictors of increased likelihood of poor outcome after MT (Alexandre et al., 2024a,b). These results suggest that achieving functional independence or favorable outcome through endovascular therapy may be particularly challenging in older patients presenting with a larger infarct core. Several potential confounding factors, including stroke etiology, onset-to-reperfusion time, and the use of intravenous thrombolysis prior to MT, did not show a significant association with worse outcomes. This lack of association may be attributed to differences in sample size and the characteristics of the study population. In our study, the significant improvement in net reclassification and integrated discrimination observed upon adding IFCI to models containing conventional risk factors underscores its incremental prognostic value. While variables like age, baseline NIHSS score, infarct core, and recanalization status remain cornerstone predictors, the IFCI provides additional biological information not captured by these clinical and imaging parameters.

Although the causal relationship between IFCI and poor outcomes post-MT has not been fully elucidated, the pathophysiological processes represented by its constituent factors may explain this phenomenon. The SII, an innovative biomarker of local immune response and systemic inflammation derived from neutrophil, lymphocyte, and platelet counts, has been reported to induce neuronal damage, blood-brain barrier dysfunction, and impaired neuroplasticity (Cicchese et al., 2018; Wang et al., 2016). These processes can contribute to larger infarct volumes, hemorrhagic transformation, and poor functional outcomes following reperfusion (Wang et al., 2025). In a retrospective cohort study of 379 stroke patients undergoing MT for large vessel occlusion, a higher admission SII showed an independent, dose-dependent association with increased sICH risk, with the highest tertile having a 3.38-fold greater risk than the lowest (Yang et al., 2022). Similarly, in another cohort of 190 patients treated with intravenous thrombolysis, elevated SII levels within 24 h of admission were significantly associated with unfavorable functional outcomes and higher risks of recurrence or mortality (Ma et al., 2023). Concurrently, the activation of often-systemic fibrotic mechanisms may indicate not only comorbidities such as subclinical liver disease but also a generalized predisposition toward excessive extracellular matrix deposition and tissue scarring (Ruiz-Villalba and Pardo-Saganta, 2025). The FIB-4 index, initially a surrogate for hepatic fibrosis, has been linked to an increased risk of symptomatic intracranial hemorrhage (Xu et al., 2024) after MT and worse outcomes in hypertrophic cardiomyopathy patients (Abdu et al., 2024). This suggests its utility as a marker of systemic fibrotic activity and potentially reduced organ reserve. Accumulating evidence suggests that liver fibrosis indices correlate with systemic inflammatory burden, endothelial dysfunction, and metabolic disturbances, all of which are implicated in blood–brain barrier disruption, secondary injury, and impaired neuroplasticity following reperfusion (Zhang et al., 2025). Furthermore, the concept of an ‘immune-liver-brain axis' has emerged, whereby liver-derived inflammatory and fibrotic signals modulate neuroinflammation and tissue remodeling after acute brain injury (Yang et al., 2024). These observations raise the possibility that FIB-4 may serve as a marker of systemic vulnerability that compromises recovery after stroke. However, direct evidence linking FIB-4 to intracranial fibrosis or neural repair mechanisms is currently lacking; thus, the proposed pathways remain speculative and warrant further investigation in mechanistic studies. The IFCI likely identifies patients experiencing a “double-hit”: a state of excessive inflammation combined with a weakened ability to repair tissue. This vulnerability becomes pivotal after MT. Even when blood flow is restored, setting the stage for healing, the fragile balance in the tissue environment can be tipped against repair by these ongoing processes (Choi et al., 2023).

Our study has several limitations that warrant consideration. Firstly, its retrospective design introduces potential biases in patient selection and data collection. The exclusion of patients with missing laboratory data, chronic inflammatory conditions, or significant pre-stroke disability may limit the generalizability of our findings to the broader stroke population. Secondly, while we adjusted for a wide array of confounders, residual confounding from unmeasured factors (e.g., specific medications, detailed socio-economic status, or genetic predispositions) cannot be excluded. Thirdly, the mechanistic links between IFCI, brain inflammation, and fibrosis remain speculative based on association; prospective studies incorporating serial biomarker measurements and advanced neuroimaging are needed to elucidate the causal pathways. Finally, the IFCI was derived and tested within the same cohort without external validation, which may introduce overfitting and optimistic performance estimates. Therefore, our findings should be considered hypothesis-generating, and the predictive accuracy of the IFCI requires confirmation in independent, prospective multicenter cohorts.

In conclusion, this study introduces the IFCI as a novel, readily available, and powerful prognostic biomarker for patients undergoing MT for acute ischemic stroke. Future prospective and multicenter studies are warranted to validate these findings, refine the index, and explore its utility in guiding targeted therapeutic interventions to improve outcomes in the era of endovascular reperfusion therapy.

## Data Availability

The raw data supporting the conclusions of this article will be made available by the authors, without undue reservation.
